# Book Review: Young, Gifted and Black: Meet 52 Black Heroes From Past and Present

**DOI:** 10.3389/fped.2018.00208

**Published:** 2018-08-16

**Authors:** Chris Fradkin

**Affiliations:** ^1^Departamento de Psicologia, Pontifícia Universidade Católica do Rio de Janeiro, Rio de Janeiro, Brazil; ^2^Psychological Sciences, University of California, Merced, Merced, CA, United States

**Keywords:** African, biography, children, role models, change-makers

A key to children's health, both physically and psychosocially, is a sense of who they are and where they come from (1). A sense of roots and cultural backstory is especially critical for black children, who endure disproportionately higher rates of poverty (2, 3) and social rejection (4, 5). For this group, role models can empower; they can instill a sense of self (6). Unfortunately, in children's literature, there is an underrepresentation of black and other people of color (7), and thereby an underrepresentation of culturally compatible role models. To this end, in *Young, Gifted and Black* (8), author Jamia Wilson has assembled 52 black heroes, to remind black children of their history, and to encourage them to strive for their potential.

The volume includes Mary Seacole (heroine of the Crimean War), Matthew Henson (Arctic explorer), Ava DuVernay (first black female film director to win a Golden Globe Award and Best Director at the Sundance 2012 Film Festival), Bessie Coleman (first African American and Native American to stage a public flight), Nina Simone (singer/songwriter/Civil Rights activist), Jean-Michel Basquiat (artist), Nelson Mandela (anti-apartheid revolutionary), Barack and Michele Obama (first African American president and first lady), Cathy Freeman (champion sprinter), Malorie Blackman (U.K. Children's Laureate), Chimananda Ngozi Adiche (award winning author), Jesse Owens (four-time Olympic gold medalist), Josephine Baker (singer/entertainer), Serena and Venus Williams (tennis champions), Sidney Poitier (first black actor to win an Academy Award), Oprah Winfrey (entertainer/entrepreneur), Martin Luther King (Nobel Prize winning civil rights leader), and 33 others. While the focus is on twentieth and twenty-first century change-makers, a balance is sustained by the inclusion of such nineteenth century figures as Harriet Tubman (conductor on the Underground Railroad) and George Washington Carver (ground-breaking agriculturist).

Among the pantheon of characters profiled in the book, there is one unifying quality: their resilience. Resilience has been defined as the quality of an individual or group that enables them to “overcome situations of suffering and adversity with personal or collective strengthening…” [(9), p. 408]. Throughout the book, the author underscores this point. For example, in the bio of writer Marjorie Blackman, the author informs us that the writer's first book, *Not So Stupid*, was rejected by over 80 publishers; but, nonetheless, the writer persevered and it was published. Likewise, the author tells us that agriculturalist George Washington Carver was “rejected from college due to discrimination” [(8), p. 12]; but he too persevered and became the first black student and black teacher at Iowa State College. In describing Olympic gold medalist Cathy Freeman, the author tells us how Freeman rose from poverty and racial discrimination and how her persistence paved the way to the Olympics. And in her piece on Harriet Tubman, the author notes how Tubman, after receiving a beating from an overseer managed to escape, but returned 19 times to lead her fellow slaves to safety. The ability to rise above adversity and achieve one's wildest dreams is the glue that binds these characters together. In doing so it makes the impact of this book much greater than the sum of its parts.

Grounding the book are its stylish illustrations. Working from a pallet of earth-toned gold and green and blue, illustrator Andrea Pippins has created full page illustrations on top of which the text is overlaid. There is a consistency of tone that weaves its way from page to page that provides comfort and engagement to the reader. In lieu of a table of contents, there is a “Hall of Fame” page, which has a photo of each hero on a baseball trading card, with a banner with their name and page number. The biographies are simple single-page vignettes. The flow from page to page is seamless.

With a title from the Nina Simone song, “To Be Young, Gifted and Black,” the author has curated a collection of historic black figures, which is accessible for children seven years and upwards. In the preface, the author and illustrator call the book a “love letter” to their ancestors and “to the next generation of black change-makers” [(8), p. 1]. They stress the importance of black history as a tool for the empowerment of black children. For many black children, this book may be a lifeline, with its variety of sung and unsung heroes. For future scientists and doctors and future activists, this book may be the spark that helps them rise to their potential.

## Author contributions

The author confirms being the sole contributor of this work and approved it for publication.

### Conflict of interest statement

The author declares that the research was conducted in the absence of any commercial or financial relationships that could be construed as a potential conflict of interest.
